# Event and Comment

**Published:** 1912-01-15

**Authors:** 


### DENTAL REGISTER.

Vol. LXVI. January 15, 1912. No. 1

### EVENT AND COMMENT.

TAGGART PATENT SUIT REVIVED.—In our December issue we commented on the fact that the Taggart patent suit had been decided against Taggart by the United States District Court in Washington. It now seems that the case has been reopened in the same court, on the grounds that Taggart's lawyers were not given sufficient notice of the time set for the hearing and so were unprepared to establish their testimony. The case will be tried sometime in January.

DEATH OF DR. SAFFORD G. PERRY. Dr. Perry died at his home in New York City Dec. 22nd, 1911. He has been for many years a sort of Dean to the profession in New York City and because of his high moral and professional character he is well known all over the country, either personally or through his extensive contributions to our literature. Because of his death the banquet planned in honor of Dr. W. W. Walker, has been indefinitely postponed. Probably no dentist since Dr. W. H. Atkinson, has had so strong an influence for high professional ideals in his own city. He was not only a scholar and professional man of the highest attainments, but he was a polished gentleman of the ideal type, strong and self-possessed, but kind and affectionate with all. His death will be a severe loss to the profession of New York City and the whole country.

DR. C. M. WRIGHT. The many friends of Dr. Wright will be grieved to learn of his sudden and untimely death from a cerebral hemorrhage on the 15th of November. A sketch of his life appears elsewhere in this issue. He was one of those lovable men that made his acquaintance a pleasure, and his intimate friendship was most highly prized by all who knew him well enough to appreciate his generous and happy disposition. He was a most honorable and ethical practitioner and a student of both the art and science of his profession. He was well grounded in literary principles and wrote good english in a most fascinating style. He had a keen sense of the humorous and his serious productions were often illuminated and made forceful by his witty statements. He made a good name for himself in both Europe and America as a practitioner and was the prime mover in organizing the present American Dental Society in Europe. He was never a professional politician, but a frequent attendant on dental societies, and always contributed his share in the discussions of all scientific subjects. For many years he has taught Physiology and Histology in the Ohio College of Dental Surgery, and the many students, who have sat under his presentation of these, to most dental students uninteresting subjects, will always be grateful that they had received their instruction from one who had the capacity to make these subjects interesting. Dr. Wright was a great and good friend and we shall all miss him from our social and professional gatherings; but his life will go on as a cherished memory in the minds of a host of admiring friends whom he has better prepared for happy and useful lives because of his kind and loving disposition and his broad knowledge. Who can estimate or value the influence of such a man? His very near friends will miss him most, and in some measure, out of their great sorrow they will be comforted by the memory of innumerable noble and affectionate associations, but the dental profession has also suffered an almost irreparable loss. We extend our sincere sympathy to his family.

REORGANIZING THE NATIONAL ASSOCIATION. The resolutions below were unamimously adopted by the Ohio State Dental Society at its annual meeting December 6th, 1911. This is the first state society to take a definite action of any encouraging character. It will be noted that the action is tentative and somewhat guarded, in that only two thirds of the entire membership is pledged and then only for a term of two years. This is probably all that can now be expected of any society and perhaps this will serve to try out the experiment without hazarding the welfare of the state and local societies irreparably. The local societies by such an agreement throw the responsibility of demonstrating the practicability and desirability of the new plan on the national organization, which will have to make good in two years. We have hoped for a complete and permanent affiliation, and now hope the experiment may result in the development of a confidential and permanent union.

Whereas, "The National Dental Association has tentatively adopted a constitution, following the plan of the American Medical Association, providing for State and Territorial Dental Societies becoming constituent members thereof and entitled to a proportionate representation in the House of Delegates, when two thirds of the membership of any State or Territorial Society is officially reported to the National as members; therefore, be it

Resolved, That the Ohio State Dental Society recognizes the need of a larger and more effective National organization and endorses the proposed plan as one which should have the support of all State and Territorial Societies; therefore, be it further

Resolved, That the Ohio State Dental Society in annual session December 5, 6, 7, 1911, officially expresses a desire to become affiliated with the National Dental Association and pledges at least two thirds of its membership for a period of two years, beginning in 1913, in accordance with the aforesaid constitution."

It is to be hoped that this matter may receive the serious consideration of every State and District Society before the next meeting of the National. The Northeastern Dental Society of New England States, has voted to become affiliated providing it is eligible and the New England State Societies approve. We see no reason why District or Sectional Societies should not so affiliate, as many of these are really stronger than the State Societies and do more active work.

CEMENT SITUATION. We are printing in this issue a most valuable paper on the present status of the various forms and manufactures of cement used in filling teeth. The article is written by Dr. W. V-B. Ames, who probably knows the cement problem as well as any other man in the country, as he has spent many years in the manufacture of cements and as a practitioner of years he had abundant opportunity to study them from the clinical standpoint. The paper is full of real information and suggestions that all dentists should know about that we may make intelligent use and also be prepared to make intelligent observations which may so enrich our experience that we shall someday produce the ideal cement filling material. The importance and timeliness of the paper justifies our reprinting it in full, although it is quite a long paper.

